# No need for a gold-standard test: on the mining of diagnostic test performance indices merely based on the distribution of the test value

**DOI:** 10.1186/s12874-023-01841-8

**Published:** 2023-01-30

**Authors:** Farrokh Habibzadeh, Hooman Roozbehi

**Affiliations:** 1 Global Virus Network, Middle East Region, Shiraz, Iran; 2 R&D Department, PIHO, Shiraz, Iran; 3 Peyvand Clinical Laboratory, Shiraz, Iran

**Keywords:** Diagnostic test, Data mining, Statistical methods, Biomarkers, Classification and taxonomy, Hepatitis

## Abstract

**Background:**

Diagnostic tests are important in clinical medicine. To determine the test performance indices — test sensitivity, specificity, likelihood ratio, predictive values, etc. — the test results should be compared against a gold-standard test. Herein, a technique is presented through which the aforementioned indices can be computed merely based on the shape of the probability distribution of the test results, presuming an educated guess.

**Methods:**

We present the application of the technique to the probability distribution of hepatitis B surface antigen measured in a group of people in Shiraz, southern Iran. We assumed that the distribution had two latent subpopulations — one for those without the disease, and another for those with the disease. We used a nonlinear curve fitting technique to figure out the parameters of these two latent populations based on which we calculated the performance indices.

**Results:**

The model could explain > 99% of the variance observed. The results were in good agreement with those obtained from other studies.

**Conclusion:**

We concluded that if we have an appropriate educated guess about the distributions of test results in the population with and without the disease, we may harvest the test performance indices merely based on the probability distribution of the test value without need for a gold standard. The method is particularly suitable for conditions where there is no gold standard or the gold standard is not readily available.

**Supplementary Information:**

The online version contains supplementary material available at 10.1186/s12874-023-01841-8.

## Background

Diagnostic tests are important means for the diagnosis of diseases. The reference range of a given marker, the test sensitivity (*Se*, the probability that a diseased person becomes test-positive) and specificity (*Sp*, the probability that a disease-free person becomes test-negative) are important test performance characteristics [1]. Positive and negative likelihood ratios (*LR*s) are other test performance indices used in clinical decision making [2]. Depending on the prior probability (prevalence, if no other information is available) of the disease (*pr*), positive (*PPV*) and negative (*NPV*) predictive values (the probabilities that a person with a positive and negative test results has the disease or not, respectively) are two other important test performance indices very useful for clinicians. Area under the receiver operating characteristic (ROC) curve (AUC) and number needed to misdiagnose (*NNM*) are other indices [3, 4].

No matter whether the test result is dichotomous (binary results, positive or negative) or continuous (where we need to use a cut-off value [also depending on the *pr*] to dichotomize the result) [4], measuring all the above-mentioned indices requires comparing our test results against the results of a gold-standard test. For certain disease conditions such as prostate cancer, we have a well-defined pathological definition of the disease and the gold-standard test is thus available. There is however, no gold-standard test for the diagnosis of some diseases, as an example, latent tuberculosis infection [5]. Hypertension is another example — it is in fact not a well-defined disease; we just know that those with higher blood pressure carry a higher risk of mortality and morbidity and thus we redefine the definition of hypertension periodically to minimize the risk incurred [6]. Sometimes, there is a gold standard, but it is invasive and costly or out of reach of many people, for example, pulmonary angiography for the diagnosis of pulmonary emboli [7]. Herein, we would like to present a method that can possibly compute the above-mentioned test performance indices merely based on the shape of the test results distribution, without any need for a gold-standard test. We also present the results of application of the method to a dataset of hepatitis B surface antigen (HBs Ag) measured in a representative sample of people residing in Shiraz, southern Iran.

### Theoretical background

**Fig. 1:**
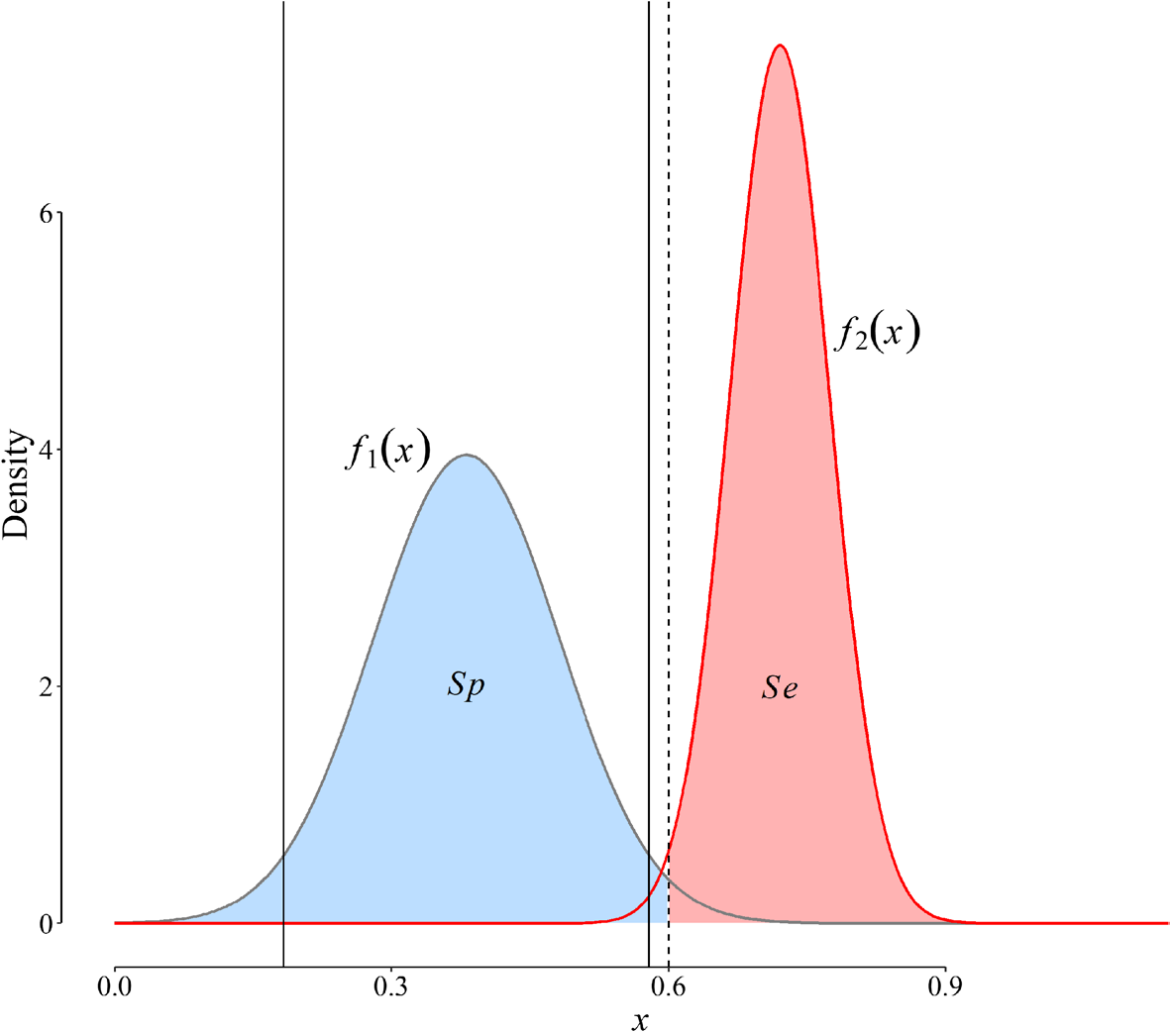
Probability distribution of a diagnostic marker (*x*) with continuous values (arbitrary unit) in disease-free (gray curve) and diseased (red curve) populations. Vertical solid lines outline the reference range of the marker values in the disease-free population. Any value equal to or greater than the cut-off value (the vertical dashed line) is considered test-positive; otherwise, it is test-negative. The pink area is equivalent to the sensitivity (*Se*); the blue area, specificity (*Sp*) [4]. Note that the curves are density functions (area under each curve is equal to 1)

Suppose that we know the probability distribution of a diagnostic marker in a group of disease-free and diseased people in a representative sample of a population (Fig. 1).

Knowing the distribution of the marker in disease-free people (gray curve, Fig. 1), we can easily determine the reference range of the marker, commonly defined as the interval between the 2.5^th^ and 97.5^th^ percentiles of the distribution of the marker (the interval between the vertical solid lines, Fig. 1) in a healthy population [8].

Let set a cut-off value of *d* (the vertical dashed line, Fig. 1). Then, any test value ≥ *d* is considered a positive test result (*T* ^+^), and according to the definition, the *Se* is [4]:

1 $$Se(d)=\int\limits_{d}^{{+\infty }} {{f_2}(x)\,dx}$$

where *f*_2_(*x*) is the probability density function of the marker distribution in diseased people (Fig. 1) [4]. In a similar way, if *f*_1_(*x*) is the probability density function of the marker distribution in the disease-free people (Fig. 1), it can be shown that the *Sp* of the marker is [4]:

2 $$Sp(d)=\int\limits_{{ - \infty }}^{d} {{f_1}(x)\,dx}$$

There is a trade-off between the test *Se* and *Sp*. Given the test *Se* and *Sp* corresponding to each cut-off value, we can construct an ROC curve which is a graphical representation of this trade-off [4]. Knowing the probability distributions ( *f*_1_ and *f*_2_, Fig. 1), we can also compute the likelihood ratios (*LRs*) for a certain value of the marker, say *x = r* as follows [2]:

3 $$LR(r)=\frac{{P\left( {x=r|{D^+}} \right)}}{{P\left( {x=r|{D^ - }} \right)}}=\frac{{{f_2}(r)}}{{{f_1}(r)}}$$

where *D* ^+^ and *D* ^–^ represent presence and absence of the disease. We may also calculate the *LR* for a range of the marker value, say for values between *s* and *r*, using the Eq. [2]:

4 $$LR(\Delta )=\frac{{P\left( {s \leqslant x<r|{D^+}} \right)}}{{P\left( {s \leqslant x<r|{D^ - }} \right)}}= - \frac{{Se(r) - Se(s)}}{{Sp(r) - Sp(s)}}$$

and for a positive and negative test results [2], assuming a cut-off value of *d*:

5 $$\begin{gathered} LR(+)=\frac{{P\left( {x \geqslant d|{D^+}} \right)}}{{P\left( {x \geqslant d|{D^ - }} \right)}}=\frac{{Se(d)}}{{1 - Sp(d)}} \hfill \\ LR( - )=\frac{{P\left( {x<d|{D^+}} \right)}}{{P\left( {x<d|{D^ - }} \right)}}=\frac{{1 - Se(d)}}{{Sp(d)}} \hfill \\ \end{gathered}$$

**Fig. 2:**
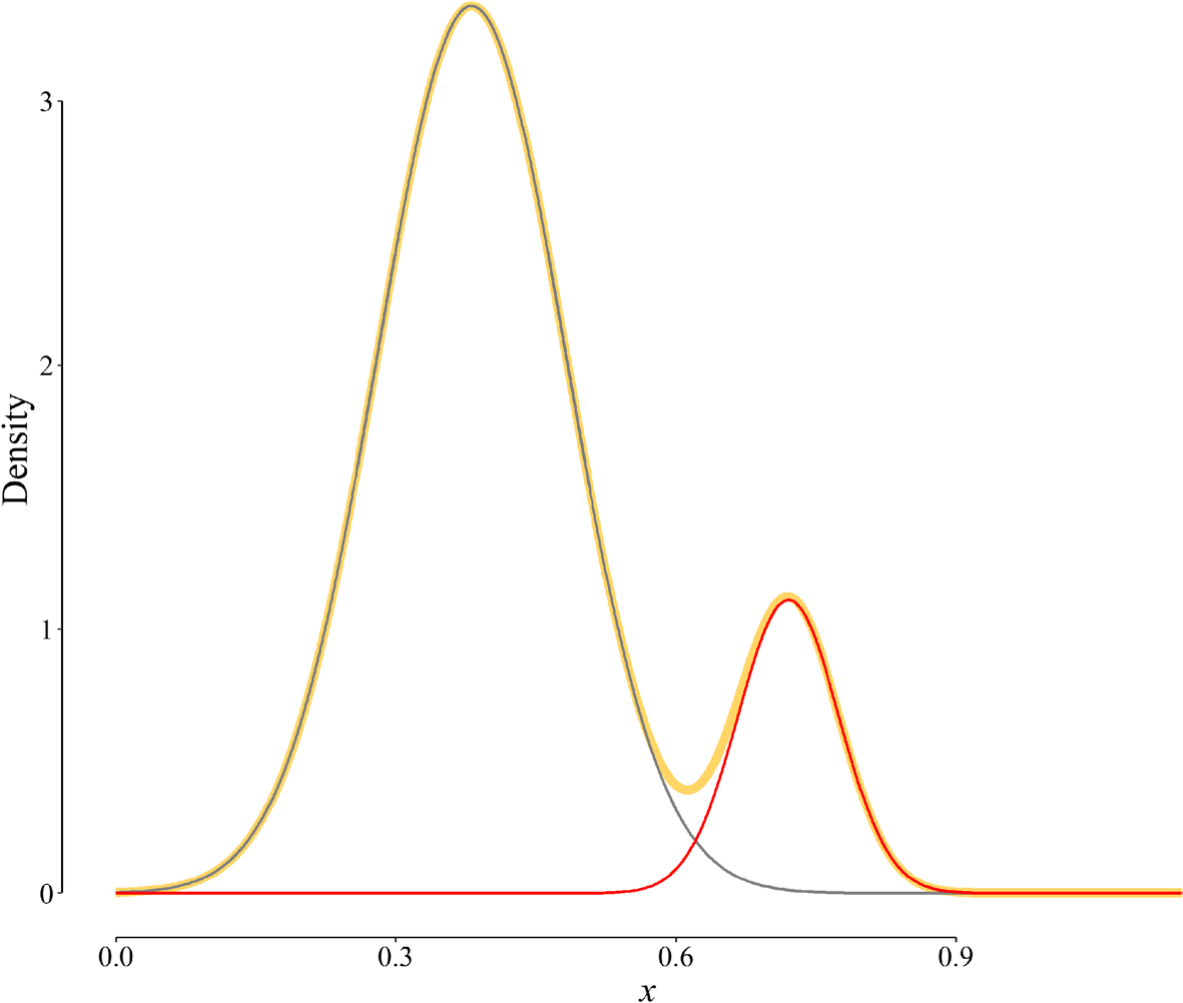
Probability distribution of the marker in the general population (yellow curve). The distribution was computed by mixing the two components using a weight of 0.85 for disease-free and 0.15 (i.e., *pr*) for diseased distributions (Eq. 6).

Using the theory of finite mixture model, we may combine the two above-said distributions of the marker in the disease-free and diseased populations with different weights to construct the distribution of the marker in the general population [9]. For example, if we combine the two distributions with weights of 0.85 and 0.15 (corresponding to a disease *pr* of 15%), we would compute the probability distribution of the marker in the general population (Fig. 2, the yellow curve) using the following equation:

6 $$Density=0.85\,{f_1}(x)+0.15\,{f_2}(x)$$

#### Reversing the process

Suppose that we have the distribution of a diagnostic marker in the general population (i.e., the yellow curve, Fig. 2). If we have a biologically plausible educated guess about the number and shape of the latent subpopulations (in our example, two components of disease-free (gray curve, Fig. 2) and diseased (red curve, Fig. 2) subpopulations), we may find the subpopulations. If we succeed, we can then compute all the test performance indices, as described above. Let us examine the method through its application to the distribution of HBs Ag in a representative sample of people residing in Shiraz, southern Iran.

## Methods

### Source of data

We analyzed the HBs Ag values taken from the database of a general clinical lab in Shiraz, southern Iran. The lab performs an average of 9000 tests each day on samples taken from about 850 people referred to the lab in different health states coming from various parts of Fars province. Data were those measured in samples received between March 2019 and March 2021 using electrochemiluminescence immunoassay (Elecsys HBsAg II, cobas^®^ e 411 analyzer, Roche Diagnostics, Switzerland). The measured HBs Ag was reported as cut-off index value, equal to test signal/cut-off.

### Statistical analysis

*R* software version 4.2.0 (*R* Project for Statistical Computing) was used for data analysis. To eliminate outliers, we only included the samples having HBs Ag values between 0.05 and 1.2. Using the default values of the *R density* function, the probability density curve for the HBs Ag values was constructed. The function uses by default a Gaussian kernel, 512 bins, and a bandwidth calculated according to the Silverman’s rule [10].

### Educated guess

**Fig. 3:**
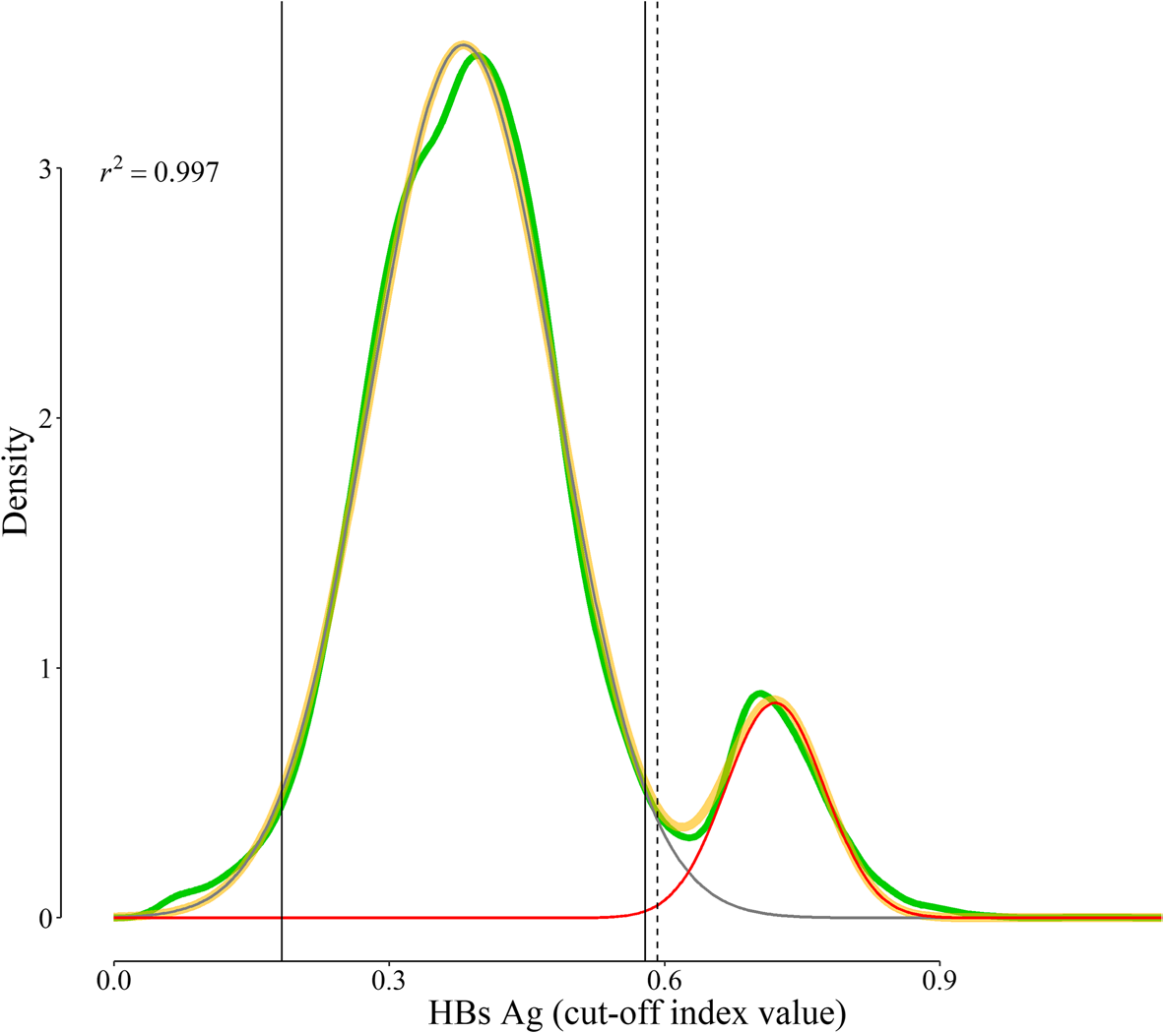
Probability distribution of HBs Ag in the studied population (green curve), and the two Gaussian components derived from curve fitting procedure. The gray curve is the distribution of HBs Ag in disease-free population; the red curve, diseased population; and the yellow curve is the superposition of the two curves, the best fit to the probability distribution of HBs Ag (the green curve)

Examination of the probability distribution of HBs Ag obtained from our dataset (green curve, Fig. 3), revealed that we may assume that there were two latent subpopulations — one for those without the disease, and another for patients with the disease. Visual examining the distribution of HBs Ag implied that it might be a mixture of at least two normally distributed latent subpopulations. We used *fviz_nbclust* function from *factoextra R* package and *clara* function from *cluster* package to determine the optimal number of latent subpopulations (eFig 1, Supplementary Materials), which confirmed the presumed number of two subpopulations. The functions also provided the first estimates for initializing the curve fitting function. We thus assumed a Gaussian mixture model with two components with the following parametric equation [11]:

7 $$\begin{aligned} y(x) &=\left( {1 - pr} \right)\,\mathcal{N}\left( {{\mu _1},\sigma _{1}^{2}} \right)+pr\,\mathcal{N}\left( {{\mu _2},\sigma _{2}^{2}} \right) \\ &=\frac{{\left( {1 - pr} \right)}}{{{\sigma _1}}}\varphi \left( {\frac{{x - {\mu _1}}}{{{\sigma _1}}}} \right)+\frac{{pr}}{{{\sigma _2}}}\varphi \left( {\frac{{x - {\mu _2}}}{{{\sigma _2}}}} \right) \\ \end{aligned}$$

where, *µ*_1_, *σ*_1_, *µ*_2_, and *σ*_2_ represent mean and the standard deviation (SD) of HBs Ag in the disease-free and diseased people, respectively; *pr* represents the prior probability (prevalence, if no other information is available) of the disease; and *φ* represents the probability density function of the Gaussian distribution.

A nonlinear curve fitting function (*nlsLM* from *minpack.ml* package for *R*) was used to compute the optimal values of parameters of a binormal equation (Eq. 7) best fit to the probability distribution. The function works based on the Levenberg-Marquardt nonlinear least-squares algorithm [12]. Constraints were imposed on the parameters *σ*_1_, and *σ*_2_ in Eq. 7 — they could only assume non-negative values; *pr*, the prior probability (or the prevalence) of the disease, could only assume values between 0 and 1, inclusive.

Having the distributions’ parameters, we can then calculate all the test performance indices — the reference range, and test *Se*, *Sp*, and *LR*s. Assuming a binormal distribution (Eq. 7), then Eqs. 1 and 2 become:

8 $$\begin{aligned} Se(d) &=\int\limits_{d}^{{+\infty }} {{f_2}(x)\,dx} \\ &=\frac{1}{{{\sigma _2}}}\int\limits_{d}^{{+\infty }} {\varphi \left( {\frac{{x - {\mu _2}}}{{{\sigma _2}}}} \right)} \,dx \\ &=1 - \Phi \left( {\frac{{d - {\mu _2}}}{{{\sigma _2}}}} \right) \\ \end{aligned}$$

and

9 $$\begin{aligned} Sp(d) &=\int\limits_{{ - \infty }}^{d} {{f_1}(x)\,dx} \\ &=\frac{1}{{{\sigma _1}}}\int\limits_{{ - \infty }}^{d} {\varphi \left( {\frac{{x - {\mu _1}}}{{{\sigma _1}}}} \right)} \,dx \\ &=\Phi \left( {\frac{{d - {\mu _1}}}{{{\sigma _1}}}} \right) \\ \end{aligned}$$

where Φ represents the cumulative distribution function of the standard normal distribution. We can construct the ROC curve and calculate the AUC. The prior probability of the disease (*pr*) can be directly derived from the fitting procedure. Given the *pr*, we may also calculate the *PPV* and *NPV* [11].

## Results

**Fig. 4:**
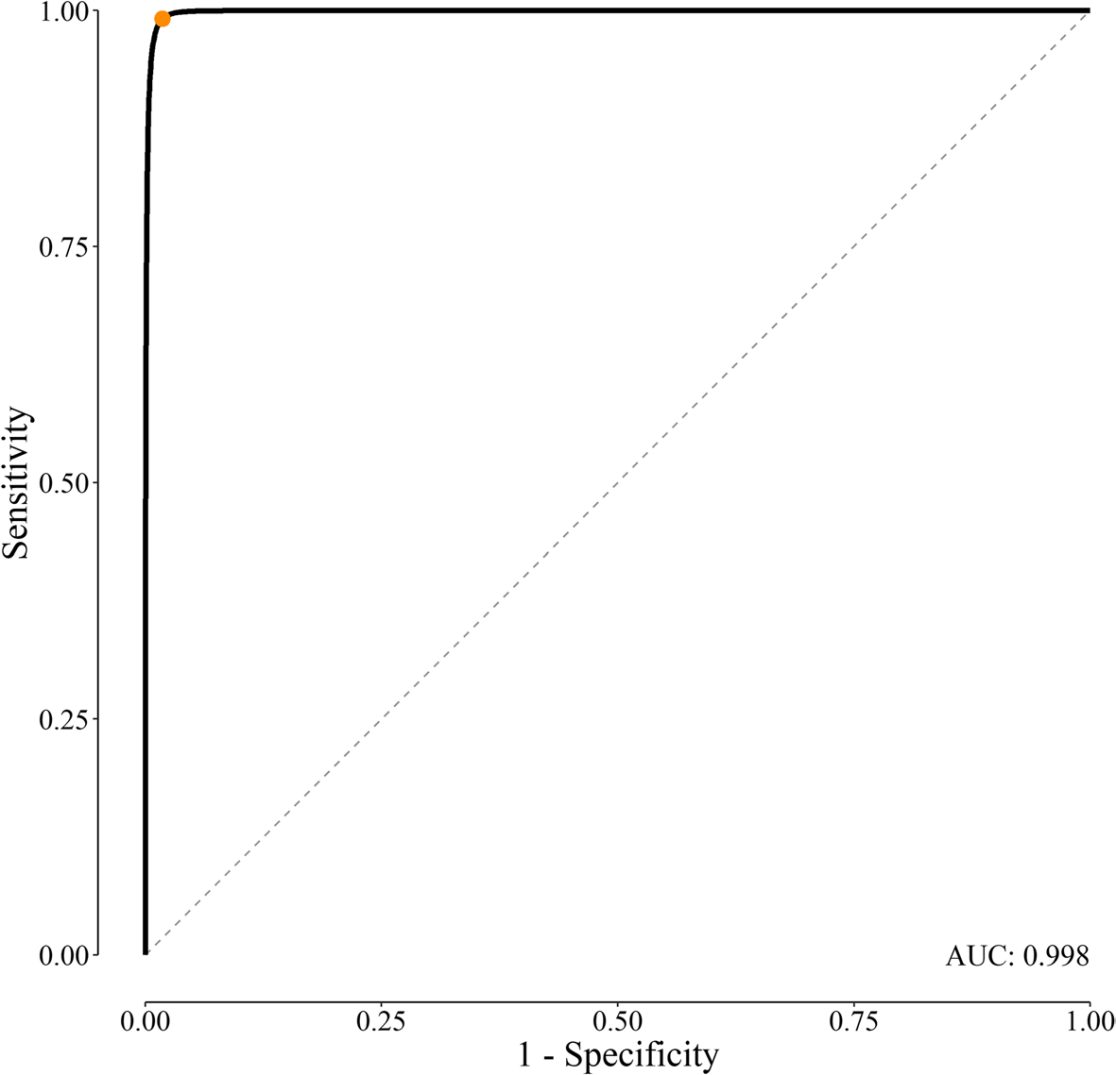
Receiver operating characteristic (ROC) curve constructed based on our dataset. The orange point corresponds to a cut-off value 0.59. The area under the curve (AUC), calculated according to DeLong, et al. [13], was 0.998

The studied dataset included 14 222 records. Excluding records with HBs Ag ≤ 0.05 (considered the lower limit of detection of the assay in our lab) or > 1.2 (leading to omission of the highest 1% of the data), left 9698 records for analyses. There were 5777 (59.6%) samples taken from females and 3921 (40.4%) from males. The mean age of study participants was 36 (SD 12) years. The probability distribution of HBs Ag had a clear bimodal distribution (green curve, Fig. 3). The technique used could correctly identify the two latent Gaussian subpopulations — one with a mean of 0.38 (SD 0.10) for disease-free people (gray curve, Fig. 3), another with a mean of 0.72 (0.05) for diseased people (red curve, Fig. 3). The reference range for HBs Ag was thus between 0.18 and 0.58 (*µ*_1_ ± 1.96 *σ*_1_, assuming the Gaussian distribution of the results; the region outlined by the two vertical solid lines, Fig. 3). The cut-off value corresponding to the maximum Youden’s J index (*Se* + *Sp* – 1) was 0.59 (vertical dashed line, Fig. 3) [4]. This cut-off value corresponds to a *Se* and *Sp* of 99.1% and 98.2%, respectively (Fig. 4).

The model could explain almost all the observed variance in the HBs Ag distribution (*r*^2^ =  0.997). The *pr* derived from the curve fitting on the subset of data (after omitting the outliers) was 11.6%, however, taking all the data into account, the *pr* corresponding to a cut-off value of 0.59 was 10.1%. The *pr* corresponding to a cut-off value of 0.9, the value suggested by the manufacturer of the diagnostic kit, was 1.2%. The cut-off corresponds to a *Se* near to zero (many false-negative results) and a *Sp* of almost 1 (no false-positive result).

**Fig. 5:**
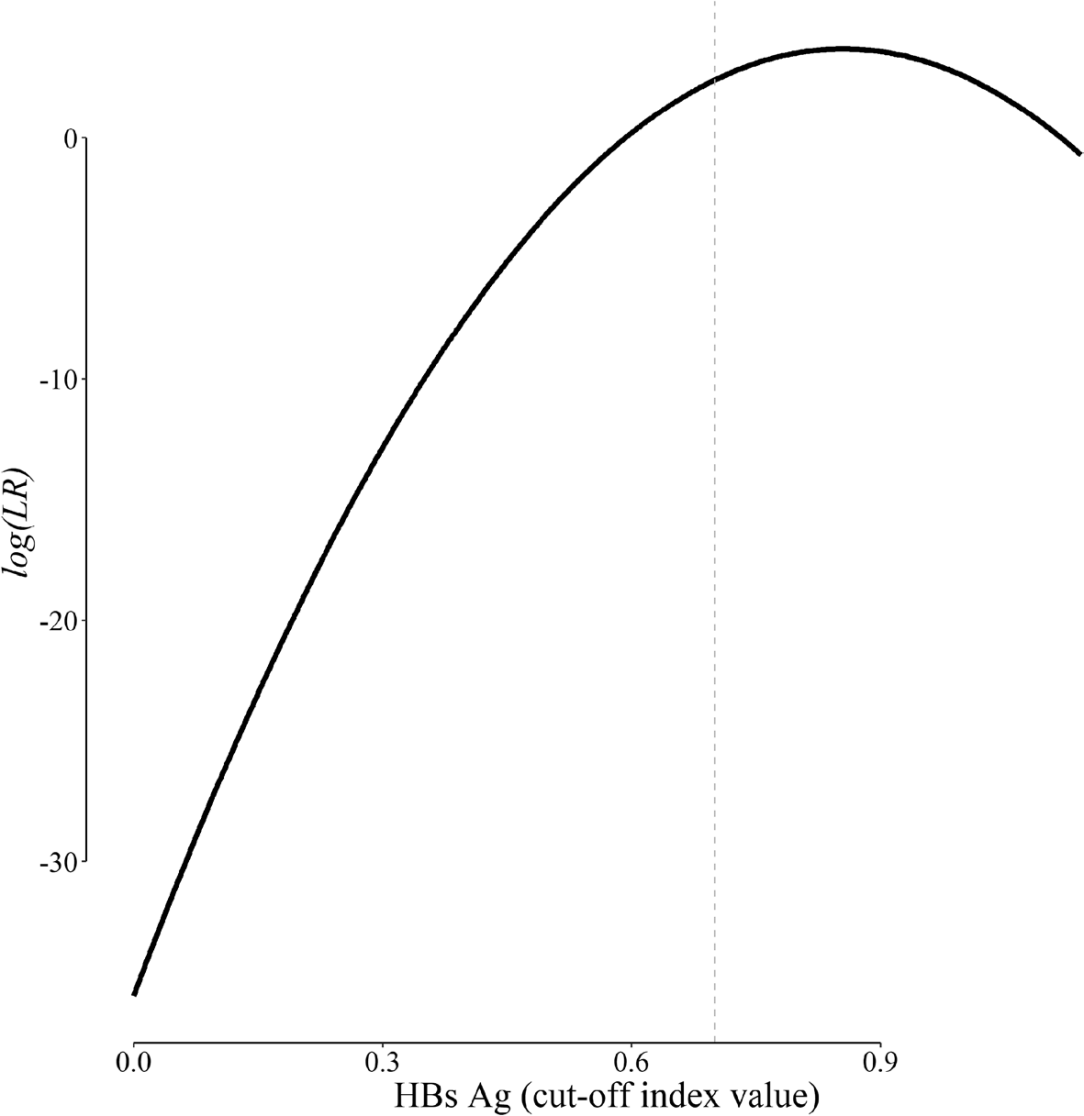
Likelihood ratio (*LR*) corresponding to each value of HBs Ag. The vertical dashed line corresponds to an HBs Ag of 0.7. Note that the y axis has a logarithmic scale

Different types of *LR*s can be calculated —﻿ for a certain HBs Ag value (Fig. 5), for a given range of HBs Ag, and for a positive or negative test result. For example, according to Eq. 3, *LR*(HBs Ag = 0.7) is:

10 $$\begin{aligned} LR(0.7) &=\frac{{P\left( {{\text{HBs Ag}}=0.7|{D^+}} \right)}}{{P\left( {{\text{HBs Ag}}=0.7|{D^ - }} \right)}}=\frac{{{f_2}(0.7)}}{{{f_1}(0.7)}} \\ &=\frac{{\frac{1}{{{\sigma _2}}}\varphi \left( {\frac{{0.7 - {\mu _2}}}{{{\sigma _2}}}} \right)}}{{\frac{1}{{{\sigma _1}}}\varphi \left( {\frac{{0.7 - {\mu _1}}}{{{\sigma _1}}}} \right)}} \\ &=\frac{{6.91}}{{0.03}} \approx 260 \\ \end{aligned}$$

which means that the likelihood of observing an HBs Ag value of 0.7 is 260 times more likely to be observed in a diseased person as compared with a disease-free person (Fig. 5) [2].

To compute the *LR* for an interval of the test results, say 0.6 ≤ HBs Ag < 0.7, we need to first calculate the *Se* and *Sp* corresponding to these values (Eq. 4), which can be done easily using Eqs. 8 and 9. The *Se* and *Sp* corresponding to a cut-off value of 0.6 is 98.7% and 98.5%, respectively; the values are 64.6% and 99.9%, respectively, for a cut-off of 0.7. Then:

11 $$\begin{aligned} LR(\Delta ) &=\frac{{P\left( {0.6 \leqslant {\text{HBs Ag}}<0.7|{D^+}} \right)}}{{P\left( {0.6 \leqslant {\text{HBs Ag}}<0.7|{D^ - }} \right)}}= - \frac{{\Delta Se}}{{\Delta Sp}} \\ &= - \frac{{64.6 - 98.7}}{{99.9 - 98.5}} \approx 24 \\ \end{aligned}$$

which means that the likelihood of observing an HBs Ag between 0.6 and 0.7 is 24 times more likely to be observed in a diseased person as compared with a disease-free person [2]. Finally, substituting the values for *Se* and *Sp* corresponding to a cut-off value of 0.59 in Eq. 5, the positive and negative *LR* are approximately 55 and 0.01, respectively [2].

Assuming a cut-off of 0.59 (which corresponds to a *pr* of 10.1% in whole population), provides a *PPV* of 86% (the probability of presence of the disease if the test is positive), *NPV* of almost 100% (the probability of absence of the disease if the test is negative), and an *NNM* of 58.

## Discussion

Using the presented method, we could harvest the test performance indices for a diagnostic test (in our example, HBs Ag) merely based on the shape of the frequency distribution of a biomarker with an acceptable accuracy, provided that we have an educated guess about frequency distributions of the test values in those with and without the disease. The cut-off value of 0.59 derived from our model was less than that commonly used in practice. The manufacturer suggests that HBs Ag values < 0.9 be interpreted as non-reactive (negative test result); those > 1.0, positive; and those between 0.9 and 1.0, borderline or equivocal. The *pr* corresponding to a cut-off value of 0.9, the value suggested by the manufacturer of the diagnostic kit, was 1.2%, consistent with the *pr* of HBs Ag reported in various seroepidemiologic studies conducted in Shiraz, Fars province [14–16]. The cut-off corresponds to a *Se* near to zero (many false-negative results) and a *Sp* of almost 1 (no false-positive result) and it seems that the cut-off value of 0.59 derived by our model (corresponding to a *Se* and *Sp* of 99.1% and 98.2%, respectively) is more reasonable (Fig. 4).

Seroprevalence studies commonly use diagnostic tests that are not perfect — the results may be false-positive or false-negative. Therefore, the *pr* calculated is just the apparent prevalence, not the true prevalence [17]. The important thing to be noted is that the *pr* derived through the method presented in this paper gives the true prevalence, not the apparent prevalence [18], which is an advantage of the metho presented.

This *LR* corresponding to each HBs Ag value is in fact the slope of the line tangent to the ROC curve at the point corresponding to that HBs Ag. This value could not usually be calculated readily in practice because the ROC curve is typically constructed based on a finite number of discrete values — the curve is thus not differentiable and the slope of the tangent line cannot be computed [2, 4]. Finding the parameters of the distribution components ( *f*_1_ and *f*_2_) through curve fitting enables us to directly calculate the slope and thus, the *LR* (Fig. 5), which is another advantage of the method proposed.

A cut-off value of 0.59, derived by the model, gave a *PPV* of 86%, an *NPV* of almost 100%, and an *NNM* of 58, which means that in average, one out of 58 independent tests performed would be either false-positive or false-negative [3]. Given that the *NPV* is almost 100%, there would be no false-positive. Therefore, a false result is most likely false-negative.

The method has been applied to distributions of other biomarkers including the prostate-specific antigen and antibody against severe acute respiratory syndrome coronavirus 2 (SARS-CoV-2) with very good results [11, 18]. The only difference was that in previous works the variables were transformed to give a better fit result; for HBs Ag, no transformation was necessary.

The method presented heavily relies on the educated guess used in constructing the model. The shapes of the probability distributions of the latent subpopulations (not necessarily the same; they may have two completely different distributions) should be reasonable and biologically plausible. We may figure out the optimum number of latent subpopulations (as we did in our study), but the number ultimately chosen for the model should be biologically plausible too. For example, if we are going to study the distribution of hemoglobin in women, we expect to have three subpopulations — those with low (anemia), normal, and high hemoglobin concentration (polycythemia). Finally, it is important to emphasize that a model is neither correct nor wrong; it may be good or bad. A good model may be but not necessarily correct.

## Conclusion

Based on the technique presented we could compute all test performance indices with clinically acceptable accuracy merely based on the distribution of the test value without the need for a gold-standard test. This technique could be of particular importance for disease conditions where no clear pathologic definition has been provided (e.g., hypertension). A diagnostic test is technically a binary classifier. The technique presented can have a wide range of applications in many scientific fields.

## Supplementary Information

**Additional file 1:** **Figure 1. **Optimal number of clusters derived from *fviz_nbclust*. The vertical dashed line corresponds to the optimal number of clusters, here 2.

## Data Availability

All data generated or analyzed during this study as well as the R codes are included in this published article and its supplementary information files.

## Abbreviations

Se: Sensitivity
Sp: Specificity
LR: Likelihood ratio
pr: Prior probability
PPV: Positive predictive value
NPV: Negative predictive value
ROC: Receiver operating characteristic
AUC: Area under the curve
HBs Ag: Hepatitis B surface antigen
SD: Standard deviation
SARS-CoV-2: Severe acute respiratory syndrome coronavirus 2
