# Supplementary material for: No need for a gold-standard test: on the mining of diagnostic test performance indices merely based on the distribution of the test value
Source: BMC Med Res Methodol. 2023 Jan 30;23:30. doi: 10.1186/s12874-023-01841-8 (PMC9885658; doi:10.1186/s12874-023-01841-8)
Supplement: Supplementary file 1 — Additional file 1: Figure 1. Optimal number of clusters derived from fviz_nbclust. The vertical dashed line corresponds to the optimal number of clusters, here 2. [file 12874_2023_1841_MOESM1_ESM.docx]

**Supplementary Materials**


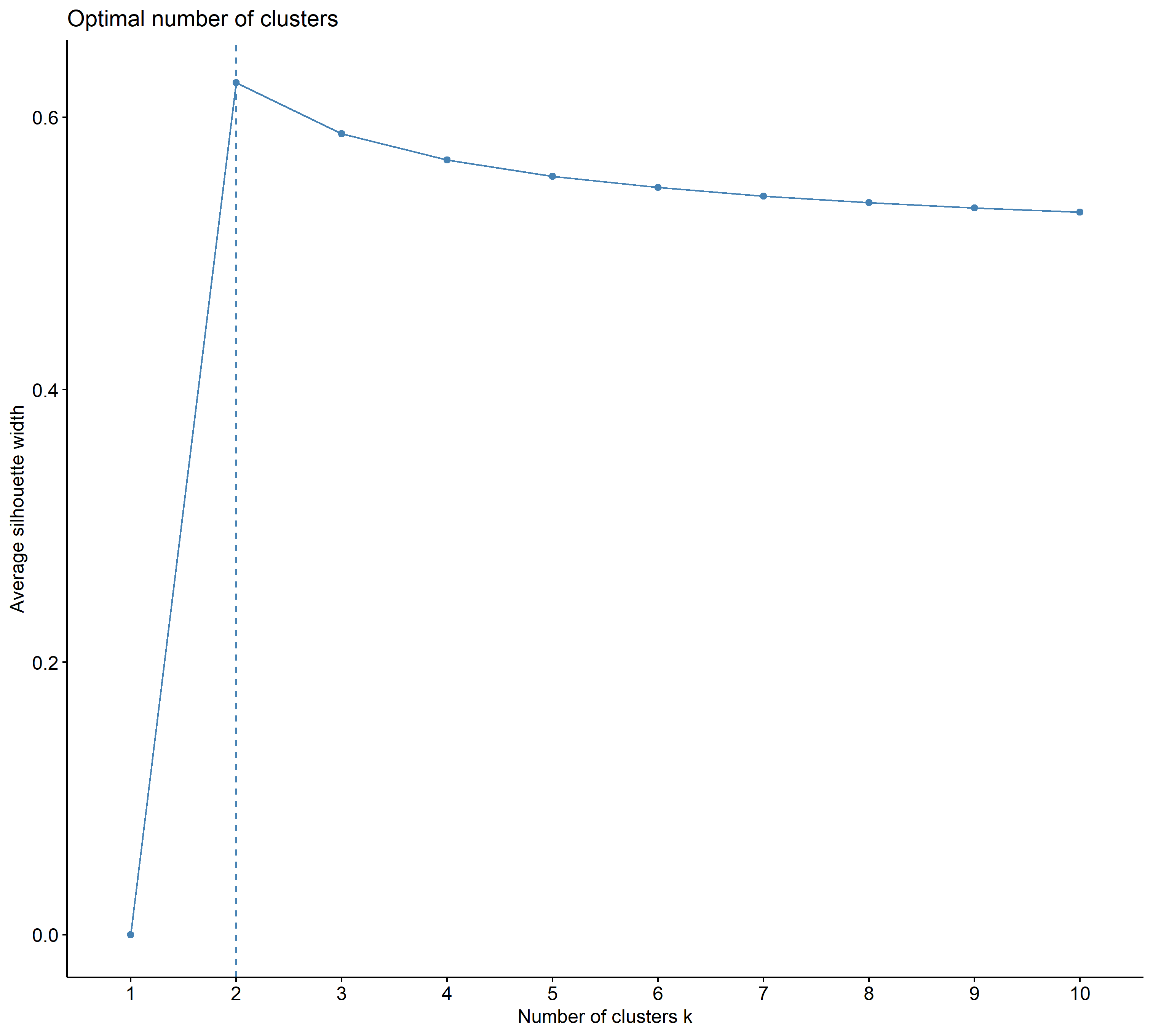


***e*Figure 1. Optimal number of clusters derived from *fviz_nbclust*.** The vertical dashed line corresponds to the optimal number of clusters, here 2.

***R* codes**

#--- Codes for determining the number of latent subpopulations. (c) Farrokh Habibzadeh, 2022

library(minpack.lm)

library("ggplot2")

library("ggh4x")

library("cluster")

library("factoextra")

#--- Normal function to be used by nonlinear curve fitting

f <- function(x, m, s)

{

return(dnorm(x, m, s))

}

#------------------------------------------------------------------

dd <- read.csv("HBSAg.csv", header=TRUE)

#--- Filter the data; removing outliers

dat <- dd[dd$HBS > 0.05 & dd$HBS <= 1.2,]

dens<-density(dat$HBS, kernel = "gaussian", na.rm = TRUE)

dens <- data.frame(x = dens$x, y = dens$y)

#--- Determining the number of clusters ----------------------------

set.seed(2022)

n <- fviz_nbclust(as.data.frame(dens$x), kmeans, method = "silhouette")$data

j <- as.integer(n[which(n$y==max(n$y)),]$clusters)

clara.res <- clara(as.data.frame(dens$x), j, samples = 50, pamLike = TRUE)

dens <- cbind(dens, clust = clara.res$cluster)

#--- First guess for parameters

st <- list(pr=0.2, m1=round(as.numeric(clara.res$medoids[1]), 2),

s1=round(sd(dens[dens$clust==1,1]), 2),

m2=round(as.numeric(clara.res$medoids[2]), 2),

s2=round(sd(dens[dens$clust==2,1]), 2))

dens<-dens[,c(1,2)]

#--- nonlinear fitting

fit <- nlsLM(dens$y ~ (1 - pr) * f(dens$x, m1, s1) + pr*f(dens$x, m2, s2),

data=dens, start=st, lower=c(0, -Inf, 0, -Inf, 0),

upper=c(1, Inf, Inf, Inf, Inf))

pr <- as.numeric(coef(fit)[1])

m1 <- as.numeric(coef(fit)[2])

s1 <- as.numeric(coef(fit)[3])

m2 <- as.numeric(coef(fit)[4])

s2 <- as.numeric(coef(fit)[5])

#--- Constructing the components

dens$nl <- (1-pr) * f(dens$x, m1, s1) #-- disease-free population

dens$dis <- pr * f(dens$x, m2, s2) #-- diseased population

#--- cut-off ----------------------------------------------------

#-- TRUE: Maximizing Youden’s index

#-- FALSE: Maximizing weighted Number Needed to Misdiagnose

Y <- TRUE

C <- 10 #-- cost of false-negative relative to false-positive result

if(Y){

C <- (1-pr)/pr #-- To maximize the Youden's index

}

d <- (m2 - m1)/s1

s <- s2/s1

cut <- (s*sqrt(2*(s^2-1)*log(s*(1-pr)/(C*pr))+d^2)-d)/(s^2-1)

cut <- m1 + cut*s1

#-- Calculating R^2

RSS <- sum(residuals(fit)^2)

TSS <- sum((dens$y - mean(dens$y))^2)

R.square <- 1 - (RSS/TSS)

ggplot(data = dens) +

geom_line(aes(x = x, y = y), color = "green3", size = 2, alpha=1)+

geom_line(aes(x = x, y = nl+dis), color = "#FFBB00", alpha=0.6, size = 2.5) +

geom_line(data = dens, aes(x = x, y = nl), color = "gray50", size = 0.8) +

geom_line(data = dens, aes(x = x, y = dis), color = "red", size = 0.8) +

geom_vline(xintercept = c(m1 - 1.96 * s1, m1 + 1.96 * s1)) +

geom_vline(xintercept = cut, linetype = "dashed") +

annotate("text", x = 0.05, y=3, size = 6, family="A",

label = paste0("italic(r)^2==", round(R.square, 3)), parse=TRUE) +

theme_classic() +

theme(text=element_text(family="A")) +

theme(legend.title = element_text("Disease"),

axis.text = element_text(size = rel(1.5), color = "black"),

axis.title = element_text(size = rel(1.9)),

aspect.ratio = 5/6,

legend.text = element_text(size = rel(0.9))

) +

labs(x = "HBs Ag (cut-off index value)", y = "Density") +

guides(x = "axis_truncated", y = "axis_truncated")
